# Supplementary material for: Maternal smoking behaviour during pregnancy and the association of Sudden Unexpected Infant Death (SUID): A retrospective cohort study of births in the United States from 2017–2021
Source: PLoS One. 2026 Mar 30;21(3):e0344554. doi: 10.1371/journal.pone.0344554 (PMC13035152; doi:10.1371/journal.pone.0344554)
Supplement: S1 Appendix — (DOCX) [file pone.0344554.s001.docx]

**S1 Appendix. DAG of maternal smoking behaviour and SUID**
