# Supplementary material for: Maternal smoking behaviour during pregnancy and the association of Sudden Unexpected Infant Death (SUID): A retrospective cohort study of births in the United States from 2017–2021
Source: PLoS One. 2026 Mar 30;21(3):e0344554. doi: 10.1371/journal.pone.0344554 (PMC13035152; doi:10.1371/journal.pone.0344554)
Supplement: S2 Table — (DOCX) [file pone.0344554.s002.docx]

**S2 Table. Additional Demographic and maternal smoking behaviours of individuals with live births in the United States, 2017 – 2021**

|  | **Maternal Smoking Behaviour Throughout Pregnancy** | | | | | | | | | | | | | |
| --- | --- | --- | --- | --- | --- | --- | --- | --- | --- | --- | --- | --- | --- | --- |
|  | **Overall** N = 16,484,245*^1^* | **nonsmoker** N = 15,222,196*^1^* | **1st trimester light** N = 1,980*^1^* | **pre-pregnancy only heavy** N = 234,259*^1^* | **pre-pregnancy only light** N = 69,620*^1^* | **smoking cessation heavy** N = 154,618*^1^* | **2nd or 3rd trimester heavy** N = 4,406*^1^* | **smoking cessation light** N = 33,362*^1^* | **discontinuous smoking heavy** N = 17,096*^1^* | **1st trimester heavy** N = 3,692*^1^* | **continuous smoking heavy** N = 664,741*^1^* | **continuous smoking light** N = 71,790*^1^* | **discontinuous smoking light** N = 4,067*^1^* | **2nd or 3rd trimester light** N = 2,418*^1^* |
| Maternal Nativity | |  |  |  |  |  |  |  |  |  |  |  |  |  |
| Born in the U.S. | 12,836,083 (78%) | 11,609,872 (76%) | 1,814 (92%) | 224,153 (96%) | 63,645 (91%) | 150,430 (97%) | 4,240 (96%) | 31,682 (95%) | 16,733 (98%) | 3,453 (94%) | 654,800 (99%) | 69,051 (96%) | 3,916 (96%) | 2,294 (95%) |
| Born outside the U.S. | 3,648,162 (22%) | 3,612,324 (24%) | 166 (8.4%) | 10,106 (4.3%) | 5,975 (8.6%) | 4,188 (2.7%) | 166 (3.8%) | 1,680 (5.0%) | 363 (2.1%) | 239 (6.5%) | 9,941 (1.5%) | 2,739 (3.8%) | 151 (3.7%) | 124 (5.1%) |
| Marital Status |  |  |  |  |  |  |  |  |  |  |  |  |  |  |
| Married | 8,761,413 (53%) | 8,408,967 (55%) | 432 (22%) | 82,707 (35%) | 23,101 (33%) | 40,883 (26%) | 1,124 (26%) | 7,361 (22%) | 4,497 (26%) | 899 (24%) | 174,603 (26%) | 15,399 (21%) | 923 (23%) | 517 (21%) |
| Unmarried | 5,807,431 (35%) | 4,928,343 (32%) | 1,467 (74%) | 144,361 (62%) | 43,747 (63%) | 108,950 (70%) | 3,137 (71%) | 24,519 (73%) | 12,295 (72%) | 2,619 (71%) | 478,876 (72%) | 54,229 (76%) | 3,040 (75%) | 1,848 (76%) |
| Missing | 1,915,401 (12%) | 1,884,886 (12%) | 81 (4.1%) | 7,191 (3.1%) | 2,772 (4.0%) | 4,785 (3.1%) | 145 (3.3%) | 1,482 (4.4%) | 304 (1.8%) | 174 (4.7%) | 11,262 (1.7%) | 2,162 (3.0%) | 104 (2.6%) | 53 (2.2%) |
| Payment Method |  |  |  |  |  |  |  |  |  |  |  |  |  |  |
| Medicaid | 6,865,612 (42%) | 5,970,722 (39%) | 1,404 (71%) | 131,662 (56%) | 39,598 (57%) | 103,341 (67%) | 3,388 (77%) | 23,563 (71%) | 12,672 (74%) | 2,558 (69%) | 516,211 (78%) | 55,576 (77%) | 3,024 (74%) | 1,893 (78%) |
| Private insurance | 8,347,307 (51%) | 8,041,542 (53%) | 447 (23%) | 91,132 (39%) | 25,724 (37%) | 43,913 (28%) | 785 (18%) | 8,094 (24%) | 3,518 (21%) | 917 (25%) | 117,549 (18%) | 12,490 (17%) | 819 (20%) | 377 (16%) |
| Self-pay | 674,389 (4.1%) | 648,916 (4.3%) | 63 (3.2%) | 3,462 (1.5%) | 1,544 (2.2%) | 2,437 (1.6%) | 125 (2.8%) | 657 (2.0%) | 369 (2.2%) | 85 (2.3%) | 14,836 (2.2%) | 1,716 (2.4%) | 106 (2.6%) | 73 (3.0%) |
| Other | 596,937 (3.6%) | 561,016 (3.7%) | 66 (3.3%) | 8,003 (3.4%) | 2,754 (4.0%) | 4,927 (3.2%) | 108 (2.5%) | 1,048 (3.1%) | 537 (3.1%) | 132 (3.6%) | 16,145 (2.4%) | 2,008 (2.8%) | 118 (2.9%) | 75 (3.1%) |
| Maternal Age |  |  |  |  |  |  |  |  |  |  |  |  |  |  |
| Under 15 years | 7,747 (<0.1%) | 7,562 (<0.1%) | 1 (<0.1%) | 31 (<0.1%) | 22 (<0.1%) | 25 (<0.1%) | 3 (<0.1%) | 15 (<0.1%) | 1 (<0.1%) | 2 (<0.1%) | 64 (<0.1%) | 19 (<0.1%) | 0 (0%) | 2 (<0.1%) |
| 15-19 years | 757,932 (4.6%) | 687,431 (4.5%) | 165 (8.3%) | 13,779 (5.9%) | 5,329 (7.7%) | 10,519 (6.8%) | 210 (4.8%) | 3,138 (9.4%) | 1,421 (8.3%) | 295 (8.0%) | 30,322 (4.6%) | 4,758 (6.6%) | 400 (9.8%) | 165 (6.8%) |
| 20-24 years | 3,123,260 (19%) | 2,780,151 (18%) | 616 (31%) | 67,393 (29%) | 21,082 (30%) | 46,664 (30%) | 1,031 (23%) | 10,653 (32%) | 5,311 (31%) | 1,078 (29%) | 167,581 (25%) | 19,692 (27%) | 1,341 (33%) | 667 (28%) |
| 25-29 years | 4,748,060 (29%) | 4,336,190 (28%) | 610 (31%) | 75,112 (32%) | 21,229 (30%) | 48,845 (32%) | 1,433 (33%) | 10,049 (30%) | 5,404 (32%) | 1,121 (30%) | 223,290 (34%) | 22,822 (32%) | 1,199 (29%) | 756 (31%) |
| 30-34 years | 4,816,275 (29%) | 4,530,920 (30%) | 380 (19%) | 51,426 (22%) | 14,416 (21%) | 31,924 (21%) | 1,085 (25%) | 6,324 (19%) | 3,326 (19%) | 753 (20%) | 158,550 (24%) | 15,858 (22%) | 752 (18%) | 561 (23%) |
| 35-39 years | 2,481,787 (15%) | 2,354,651 (15%) | 172 (8.7%) | 22,533 (9.6%) | 6,453 (9.3%) | 14,020 (9.1%) | 525 (12%) | 2,682 (8.0%) | 1,382 (8.1%) | 345 (9.3%) | 71,359 (11%) | 7,116 (9.9%) | 323 (7.9%) | 226 (9.3%) |
| 40-44 years | 513,653 (3.1%) | 490,540 (3.2%) | 36 (1.8%) | 3,836 (1.6%) | 1,044 (1.5%) | 2,561 (1.7%) | 115 (2.6%) | 478 (1.4%) | 248 (1.5%) | 94 (2.5%) | 13,136 (2.0%) | 1,475 (2.1%) | 51 (1.3%) | 39 (1.6%) |
| 45-49 years | 32,465 (0.2%) | 31,712 (0.2%) | 0 (0%) | 145 (<0.1%) | 44 (<0.1%) | 58 (<0.1%) | 3 (<0.1%) | 23 (<0.1%) | 3 (<0.1%) | 4 (0.1%) | 422 (<0.1%) | 48 (<0.1%) | 1 (<0.1%) | 2 (<0.1%) |
| 50-54 years | 3,066 (<0.1%) | 3,039 (<0.1%) | 0 (0%) | 4 (<0.1%) | 1 (<0.1%) | 2 (<0.1%) | 1 (<0.1%) | 0 (0%) | 0 (0%) | 0 (0%) | 17 (<0.1%) | 2 (<0.1%) | 0 (0%) | 0 (0%) |
| Total Pregnancy Order | | | | | | | | | | | | | | |
| 1 | 5,217,211 (32%) | 4,912,137 (32%) | 523 (26%) | 80,888 (35%) | 25,139 (36%) | 46,917 (30%) | 729 (17%) | 10,101 (30%) | 4,518 (26%) | 1,070 (29%) | 119,013 (18%) | 14,602 (20%) | 1,086 (27%) | 488 (20%) |
| 2-3 | 7,677,712 (47%) | 7,128,195 (47%) | 867 (44%) | 103,938 (44%) | 29,961 (43%) | 67,252 (43%) | 1,921 (44%) | 14,064 (42%) | 7,311 (43%) | 1,566 (42%) | 289,391 (44%) | 30,509 (42%) | 1,693 (42%) | 1,044 (43%) |
| 4-5 | 2,610,027 (16%) | 2,340,512 (15%) | 382 (19%) | 35,804 (15%) | 10,460 (15%) | 27,884 (18%) | 1,082 (25%) | 6,190 (19%) | 3,432 (20%) | 705 (19%) | 165,415 (25%) | 16,791 (23%) | 800 (20%) | 570 (24%) |
| 5+ | 979,295 (5.9%) | 841,352 (5.5%) | 208 (11%) | 13,629 (5.8%) | 4,060 (5.8%) | 12,565 (8.1%) | 674 (15%) | 3,007 (9.0%) | 1,835 (11%) | 351 (9.5%) | 90,922 (14%) | 9,888 (14%) | 488 (12%) | 316 (13%) |
| Pregnancy Weight Gain | | | | | | | | | | | | | | |
| Less than 11 pounds | 1,603,382 (9.7%) | 1,445,233 (9.5%) | 237 (12%) | 20,290 (8.7%) | 6,805 (9.8%) | 14,591 (9.4%) | 720 (16%) | 3,660 (11%) | 2,047 (12%) | 389 (11%) | 98,032 (15%) | 10,432 (15%) | 562 (14%) | 384 (16%) |
| 11 to 20 pounds | 2,950,133 (18%) | 2,739,792 (18%) | 310 (16%) | 29,587 (13%) | 10,260 (15%) | 21,390 (14%) | 773 (18%) | 5,210 (16%) | 2,391 (14%) | 497 (13%) | 125,135 (19%) | 13,666 (19%) | 685 (17%) | 437 (18%) |
| 21 to 30 pounds | 4,718,138 (29%) | 4,426,438 (29%) | 431 (22%) | 48,743 (21%) | 15,906 (23%) | 33,321 (22%) | 1,013 (23%) | 7,554 (23%) | 3,664 (21%) | 841 (23%) | 161,615 (24%) | 17,131 (24%) | 954 (23%) | 527 (22%) |
| 31 to 40 pounds | 3,943,283 (24%) | 3,680,921 (24%) | 416 (21%) | 53,373 (23%) | 16,045 (23%) | 33,987 (22%) | 857 (19%) | 7,217 (22%) | 3,584 (21%) | 807 (22%) | 130,727 (20%) | 14,031 (20%) | 847 (21%) | 471 (19%) |
| 41 to 98 pounds | 3,269,309 (20%) | 2,929,812 (19%) | 586 (30%) | 82,266 (35%) | 20,604 (30%) | 51,329 (33%) | 1,043 (24%) | 9,721 (29%) | 5,410 (32%) | 1,158 (31%) | 149,232 (22%) | 16,530 (23%) | 1,019 (25%) | 599 (25%) |
| Pre-pregnancy Body Mass Index | | | | | | | | | | | | | | |
| Underweight <18.5 | 499,350 (3.0%) | 436,268 (2.9%) | 82 (4.1%) | 8,948 (3.8%) | 2,624 (3.8%) | 7,373 (4.8%) | 206 (4.7%) | 1,529 (4.6%) | 853 (5.0%) | 151 (4.1%) | 37,470 (5.6%) | 3,530 (4.9%) | 205 (5.0%) | 111 (4.6%) |
| Normal 18.5-24.9 | 6,820,866 (41%) | 6,344,689 (42%) | 730 (37%) | 82,496 (35%) | 25,319 (36%) | 56,602 (37%) | 1,649 (37%) | 12,383 (37%) | 6,430 (38%) | 1,357 (37%) | 258,920 (39%) | 27,920 (39%) | 1,471 (36%) | 900 (37%) |
| Overweight 25.0-29.9 | 4,440,579 (27%) | 4,129,557 (27%) | 530 (27%) | 60,156 (26%) | 18,205 (26%) | 38,184 (25%) | 1,118 (25%) | 8,129 (24%) | 4,296 (25%) | 915 (25%) | 160,373 (24%) | 17,449 (24%) | 1,046 (26%) | 621 (26%) |
| Obesity I 30.0-39.9 | 2,560,389 (16%) | 2,352,708 (15%) | 332 (17%) | 41,289 (18%) | 11,943 (17%) | 26,201 (17%) | 727 (17%) | 5,707 (17%) | 2,823 (17%) | 652 (18%) | 105,399 (16%) | 11,561 (16%) | 654 (16%) | 393 (16%) |
| Obesity II 35.0-39.9 | 1,266,777 (7.7%) | 1,151,380 (7.6%) | 163 (8.2%) | 23,382 (10.0%) | 6,503 (9.3%) | 14,735 (9.5%) | 413 (9.4%) | 3,116 (9.3%) | 1,512 (8.8%) | 361 (9.8%) | 58,270 (8.8%) | 6,342 (8.8%) | 386 (9.5%) | 214 (8.9%) |
| Extreme Obesity III ≥ 40.0 | 896,284 (5.4%) | 807,594 (5.3%) | 143 (7.2%) | 17,988 (7.7%) | 5,026 (7.2%) | 11,523 (7.5%) | 293 (6.7%) | 2,498 (7.5%) | 1,182 (6.9%) | 256 (6.9%) | 44,309 (6.7%) | 4,988 (6.9%) | 305 (7.5%) | 179 (7.4%) |
| Pre-pregnancy Hypertension | | | | | | | | | | | | | | |
| No | 16,122,948 (98%) | 14,899,446 (98%) | 1,933 (98%) | 227,409 (97%) | 67,868 (97%) | 149,884 (97%) | 4,248 (96%) | 32,368 (97%) | 16,631 (97%) | 3,581 (97%) | 643,869 (97%) | 69,419 (97%) | 3,955 (97%) | 2,337 (97%) |
| Yes | 361,297 (2.2%) | 322,750 (2.1%) | 47 (2.4%) | 6,850 (2.9%) | 1,752 (2.5%) | 4,734 (3.1%) | 158 (3.6%) | 994 (3.0%) | 465 (2.7%) | 111 (3.0%) | 20,872 (3.1%) | 2,371 (3.3%) | 112 (2.8%) | 81 (3.3%) |
| Gestational Hypertension | | | | | | | | | | | | | | |
| No | 15,244,405 (92%) | 14,083,317 (93%) | 1,821 (92%) | 211,801 (90%) | 63,582 (91%) | 140,542 (91%) | 4,094 (93%) | 30,420 (91%) | 15,917 (93%) | 3,398 (92%) | 617,048 (93%) | 66,462 (93%) | 3,763 (93%) | 2,240 (93%) |
| Yes | 1,239,840 (7.5%) | 1,138,879 (7.5%) | 159 (8.0%) | 22,458 (9.6%) | 6,038 (8.7%) | 14,076 (9.1%) | 312 (7.1%) | 2,942 (8.8%) | 1,179 (6.9%) | 294 (8.0%) | 47,693 (7.2%) | 5,328 (7.4%) | 304 (7.5%) | 178 (7.4%) |
| Prior Terminations/Fetal Death | | | | | | | | | | | | | | |
| 0 | 11,990,680 (73%) | 11,201,679 (74%) | 1,261 (64%) | 155,615 (66%) | 46,948 (67%) | 98,673 (64%) | 2,684 (61%) | 21,396 (64%) | 10,728 (63%) | 2,397 (65%) | 400,633 (60%) | 44,562 (62%) | 2,569 (63%) | 1,535 (63%) |
| 1-2 | 3,928,908 (24%) | 3,539,197 (23%) | 614 (31%) | 67,256 (29%) | 19,513 (28%) | 46,618 (30%) | 1,402 (32%) | 10,001 (30%) | 5,132 (30%) | 1,057 (29%) | 213,968 (32%) | 22,218 (31%) | 1,213 (30%) | 719 (30%) |
| 3-4 | 465,419 (2.8%) | 401,018 (2.6%) | 83 (4.2%) | 9,254 (4.0%) | 2,577 (3.7%) | 7,325 (4.7%) | 239 (5.4%) | 1,559 (4.7%) | 947 (5.5%) | 187 (5.1%) | 38,011 (5.7%) | 3,865 (5.4%) | 223 (5.5%) | 131 (5.4%) |
| 4+ | 99,238 (0.6%) | 80,302 (0.5%) | 22 (1.1%) | 2,134 (0.9%) | 582 (0.8%) | 2,002 (1.3%) | 81 (1.8%) | 406 (1.2%) | 289 (1.7%) | 51 (1.4%) | 12,129 (1.8%) | 1,145 (1.6%) | 62 (1.5%) | 33 (1.4%) |
