# Supplementary figures and images for: Maternal smoking behaviour during pregnancy and the association of Sudden Unexpected Infant Death (SUID): A retrospective cohort study of births in the United States from 2017–2021
Source: PLoS One. 2026 Mar 30;21(3):e0344554. doi: 10.1371/journal.pone.0344554 (PMC13035152; doi:10.1371/journal.pone.0344554)

**S3 Appendix. Smoking trend among individuals with live birth in the U.S. from 2017 to 2021**

**
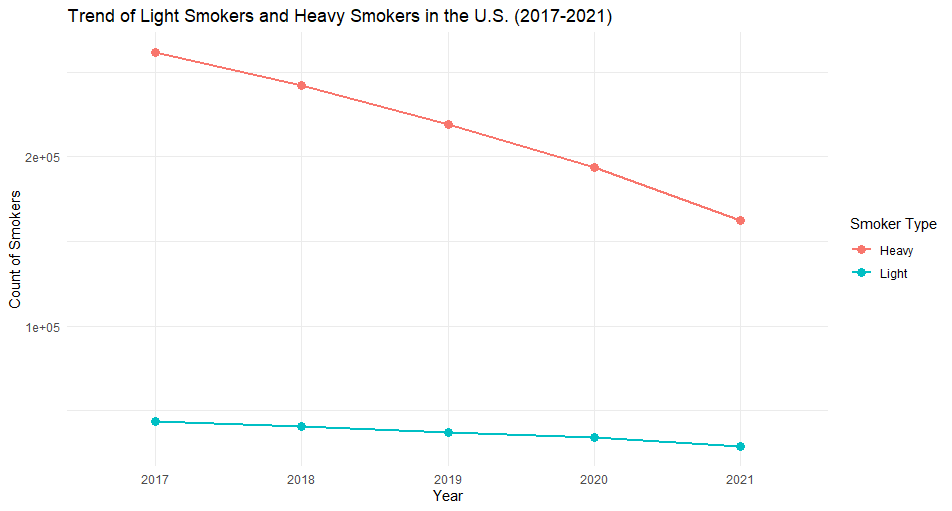
**

Supplement: S3 Appendix — (DOCX) [file pone.0344554.s003.docx]

**S4 Appendix. SUID trend among individuals with live birth in the U.S. from 2017 to 2021**

**
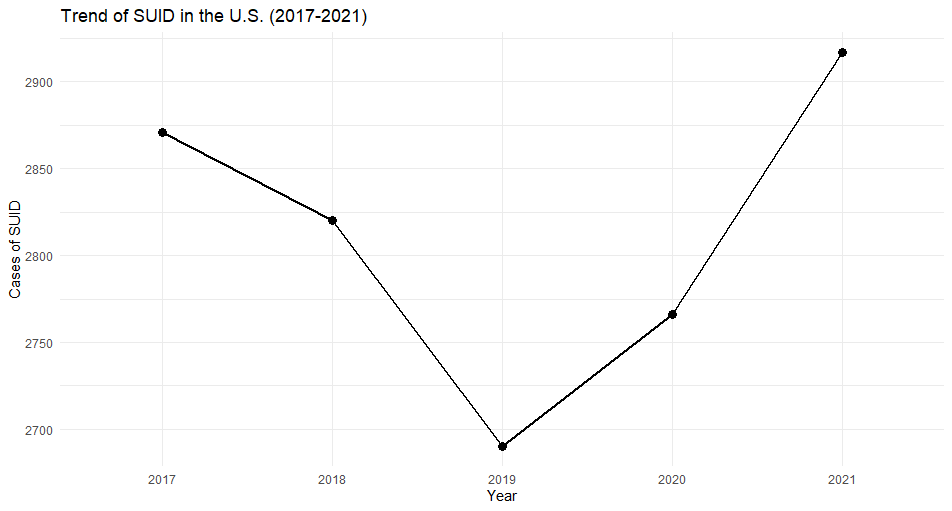
**

Supplement: S4 Appendix — (DOCX) [file pone.0344554.s004.docx]
