# Supplementary material for: Maternal smoking behaviour during pregnancy and the association of Sudden Unexpected Infant Death (SUID): A retrospective cohort study of births in the United States from 2017–2021
Source: PLoS One. 2026 Mar 30;21(3):e0344554. doi: 10.1371/journal.pone.0344554 (PMC13035152; doi:10.1371/journal.pone.0344554)
Supplement: Attachment — Submitted filename: Response to Reviewers.docx [file pone.0344554.s006.docx]

Response to Reviewers’ comments

**Reviewer #1**

Comment 1. The work presented in this manuscript is important and it is very well written. However, the title does not indicate the study setting and no indication of the type of study. Other than that, I do not believe that any editing is required except, may be in formatting and removing the title, sub title levels given within parenthesis and the numbering and titles of figures.

**Response**: Thank you. We have indicated the study setting and study type into the long title, changing it to “Maternal Smoking Behaviour During Pregnancy and the Association of Sudden Unexpected Infant Death (SUID): A Retrospective Cohort Study of Births in the United States from 2017-2021”. We have also changed the heading levels formats, and added an acknowledgement section (lines 301-304) and supporting information section (lines 406-414). Lastly, we referred to the PLOS One submission guide to ensure that our figures, table numbers, titles, and citations fit the criteria.

**Reviewer #2**

Comment 1. This is a well-written clear manuscript that adds to the literature by incorporating 'heaviness' and 'timing' of smoking during pregnancy into the SUID literature.

**Response**: Thank you.

Comment 2. There IS a paper examining the relationship between maternal obesity and SUID (<https://pubmed.ncbi.nlm.nih.gov/39073792/)...Seemingly>, obesity (which is available in the CDC dataset) should be a covariate.

**Response**: Thank you for pointing out the lack of maternal obesity indicated as covariate despite it being included in our analysis. We have indicated pregnancy body mass index as a covariate in our manuscript (lines 121), have added its distribution into the S2 Table, and added reference #18: Tanner D, et al. Maternal obesity and risk of sudden unexpected infant death. JAMA Pediatrics. 2024;178(9):906-913. doi: 10.1001/jamapediatrics.2024.2455.

Comment 3. The authors should re-order table 2, from highest to lowest aOR, and then describe findings in the results section (and abstract) going down from highest to lowest. As it is, the jumping around makes it confusing to a reader. Similarly, reorient the forest plot in the same way.

**Response**: We have reordered our findings in Crude Rate (line 168-177) and Adjusted Results (line 191-199, line 204-207 going from highest to lowest. However, regarding the forest plot and Table 2, while we understand the reviewer’s point, given that our study seeks to examine duration since smoking cessation, presenting smoking levels in a sequential way will more clearly show timing of smoking cessation and its relationship and trends with SUID. Additionally, keeping heavy and light smoker together for each smoking category will make it easier to evaluate dose response.
